# The increased prevalence of *Vibrio* species and the first reporting of *Vibrio jasicida* and *Vibrio rotiferianus* at UK shellfish sites

**DOI:** 10.1016/j.watres.2021.117942

**Published:** 2022-03-01

**Authors:** Jamie Harrison, Kathryn Nelson, Helen Morcrette, Cyril Morcrette, Joanne Preston, Luke Helmer, Richard W. Titball, Clive S. Butler, Sariqa Wagley

**Affiliations:** a Biosciences, College of life and Environmental Sciences, University of Exeter, Devon, Exeter EX4 4QD, UK; b Sussex Inshore Fisheries and Conservation Authority, 12a Riverside Business Centre, Brighton Road, Shoreham BN43 6RE, UK; c Met Office, FitzRoy Road, Devon, Exeter EX1 3PB, UK; d Institute of Marine Sciences, School of Biological Sciences, University of Portsmouth PO4 9LY, UK; e Blue Marine Foundation, Somerset House, London WC2R 1LA, UK

**Keywords:** *Vibrio* species, *Vibrio jasicida*, *Vibrio rotiferianus*, *Vibrio parahaemolyticus*, Shellfish, Sea-surface temperature

## Abstract

Warming sea-surface temperature has led to an increase in the prevalence of *Vibrio* species in marine environments. This can be observed particularly in temperate regions where conditions for their growth has become more favourable. The increased prevalence of pathogenic *Vibrio* species has resulted in a worldwide surge of Vibriosis infections in human and aquatic animals. This study uses sea-surface temperature data around the English and Welsh coastlines to identify locations where conditions for the presence and growth of *Vibrio* species is favourable. Shellfish samples collected from three locations that were experiencing an increase in sea-surface temperature were found to be positive for the presence of *Vibrio* species. We identified important aquaculture pathogens *Vibrio rotiferianus* and *Vibrio jasicida* from these sites that have not been reported in UK waters. We also isolated human pathogenic *Vibrio* species including *V. parahaemolyticus* from these sites. This paper reports the first isolation of *V. rotiferianus* and *V. jasicida* from UK shellfish and highlights a growing diversity of *Vibrio* species inhabiting British waters.

## Highlights

• SSTs in UK shellfish harvesting waters are 18–22 °C for 8 consecutive weeks.
• Increasing SSTs do not necessarily drive growth of *V. parahaemolyticus* in shellfish.
• Whole genome sequencing identifies *V. rotiferianus* and *V. jasicida* in shellfish.
• *V. jascinda* and *V. rotiferianus* show pathogenicity in insect infection model.

## 1 Introduction

The aquaculture industry provides an increasing contribution to global food supplies; however, it is faced with challenges from a changing climate, globalisation, ecological constraints, and human population changes (both growth and food preference). The UK shellfish industry is worth over £350 million annually (UK Sea Fisheries Statistics, 2019), providing an important contribution to the UK aquaculture sector and economy. The farming of molluscan shellfish in nearshore ecosystems accounts for a large portion of the shellfish industry. Molluscan shellfish are filter feeders i.e., they pass water across their gills, filtering and selecting particles for consumption, including algae and bacteria. Consequently, pathogenic bacteria, including *Vibrio* species, can accumulate to levels where the shellfish become unsafe to eat. In the United States, 76% of seafood-associated infections, between 1973 and 2006, were due to bacteria (Iwamoto et al., 2010). In previous studies, *Vibrio parahaemolyticus* levels have been shown to be up to five times higher within crab tissues than in the surrounding water (Wagley et al., 2009) indicating that in some ecological habitats, *Vibrio* species are extremely abundant within shellfish (Parveen et al., 2008; Wagley et al., 2009). There is evidence that the increased global incidence of Vibriosis (any disease caused by a *Vibrio* species) is linked to climate change, with altered patterns of bacterial survival in shellfish (Baker-Austin et al., 2013; Costello et al., 2009; Froelich and Daines, 2020; Leddin and Macrae, 2020; Lindgren et al., 2012; Martinez-Urtaza et al., 2010; Takemura et al., 2014). Consequently, global increases in sea-surface temperatures in nearshore ecosystems can drive the emergence of a range of *Vibrio* species including pathogens of humans, marine finfish, shellfish and crustaceans in regions where this has not been previously seen (Baker-Austin et al., 2013; Froelich and Daines, 2020; Lindgren et al., 2012; Takemura et al., 2014).

*Vibrio* species can cause disease in shellfish in both aquaculture settings and the natural environment. These *Vibrio* species include *V. aestuarianus, V. crassostreae* and *V. coralliilyticus* which are responsible for the sharp decline of Pacific oyster numbers (*Crassostrea gigas*), resulting in serious socioeconomic losses in Europe, New Zealand and Australia (European Commission, 2015; de Lorgeril et al. 2018; Elston et al., 2008; Goudenege et al., 2015). Members of the *V. harveyi* clade such as *V. campbelli* and *V. rotiferianus* can also cause disease in marine fish and shrimp (European Commission, 2015; Austin and Zhang, 2006; Bruto et al., 2017; de Lorgeril et al. 2018; Elston et al., 2008; Goudenege et al., 2015; Travers et al., 2015). The poleward spread of human pathogenic *Vibrio* species, such as *V. parahaemolyticus* and *V. vulnificus*, is concurrent with increasing global temperature and reduced sea surface salinity, contributing to an increase in human disease burden globally (Baker-Austin et al., 2010, 2013; Martinez-Urtaza et al., 2010). Outbreaks of Vibriosis in humans have recently been reported in regions of the world previously considered to have temperate or cold climates including Peru (during its austral winter) (Fuenzalida et al., 2007), Chile (Gonzalez-Escalona et al., 2005), Alaska (McLaughlin et al., 2005), Denmark (Dalsgaard et al., 1996), Spain (Martinez-Urtaza et al., 2005, 2018) and Pacific Northwest USA (Martinez-Urtaza et al., 2013). Collectively these findings support the hypothesis that *Vibrio*-associated diseases are increasing and are influenced by the rise in global sea temperature.

There is a strong seasonal pattern related to temperature and abundance of *Vibrio* species (Kokashvili et al., 2015; Vezzulli et al., 2016). Nearly all *Vibrio* species can grow at temperatures between 13 and 22 °C and where salinity ranges from 5 to 25 parts per thousand (ppt) (Kaspar and Tamplin, 1993). Under these conditions, most *Vibrio* species will proliferate in shellfish and this coincides with elevated disease burden (Ansede-Bermejo et al., 2010; Joseph et al., 1982; McLaughlin et al., 2005; Mead et al., 1999). In the UK, *Vibrio* species are detected in domestically grown shellfish from May – October, with peak levels occurring at the end of August and the start of September (Ford et al., 2020a; Powell et al., 2013; Wagley et al., 2009, 2008). In this study, sea-surface temperature data were analysed to identify the locations where *Vibrio* species were most likely to be found around the English and Welsh coastlines. Shellfish samples were then sourced from these areas, and from a nearby region of cooler waters. The prevalence of *Vibrio* species in shellfish from Chichester Harbour, Osea Island, Whitstable Bay and Lyme Bay in the summer of 2018 was determined using culture-based methods. Several strains isolated from these sites were genome sequenced in order to ascertain their taxonomy and investigate their genomics.

## 2 Results

### 2.1 Sea-surface temperature at different sites in England and Wales

**Fig. 1:**
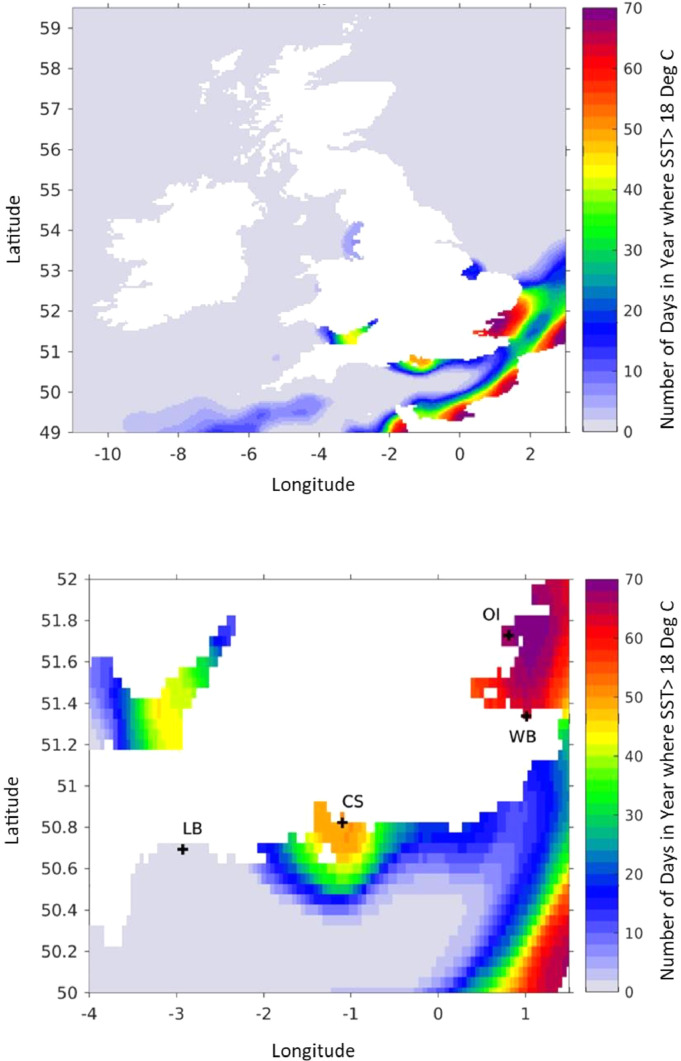
Average number of days per year when the sea-surface temperature is above 18 deg C. Fig. 1a is a focus on Britain and Ireland, while Fig. 1b is focus on southern England with the locations of Lyme Bay (LB), Chichester/Solent (CS), Osea Island (OI) and Whitstable Bay (WB) marked with crosses. Both figures are calculated as a mean over the 3 years 2015–2017 using OSTIA analyses.

**Fig. 2:**
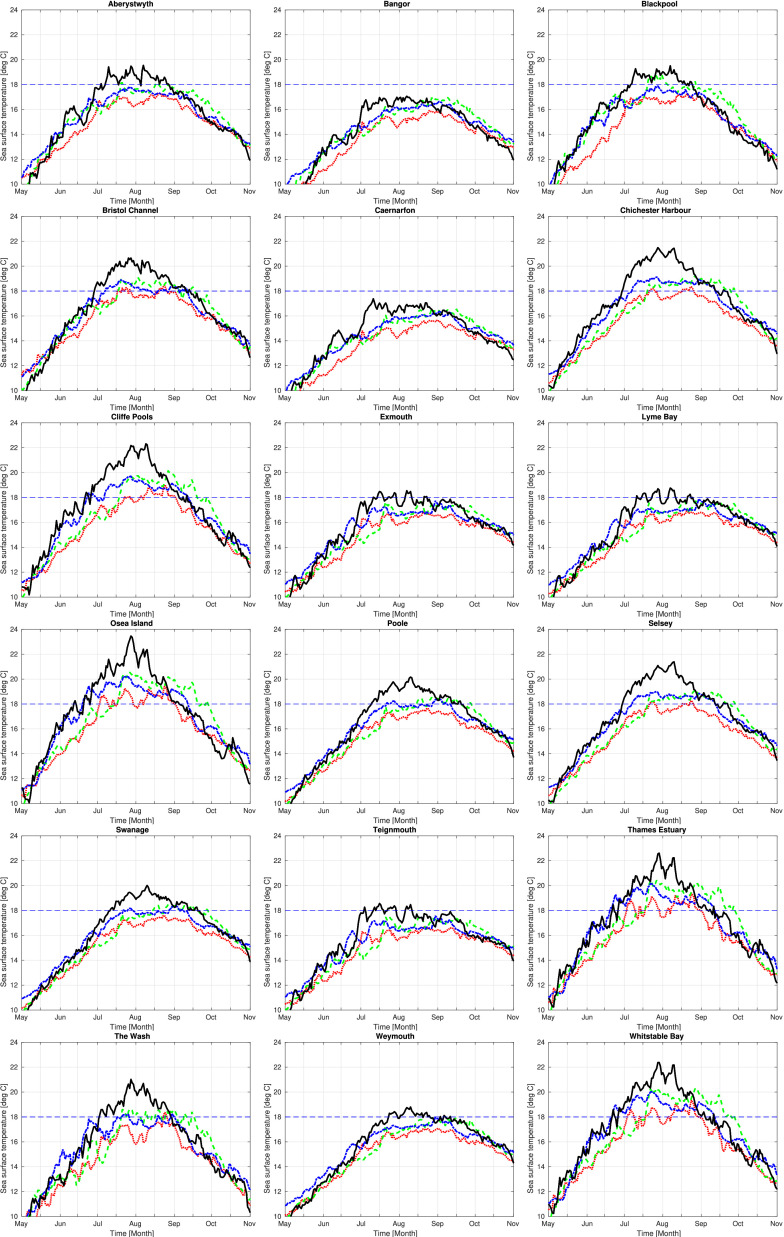
Time series of sea-surface temperature data [deg C] (at foundation depth, from OSTIA) from 2015 to 2018 in England and Wales. For 2015 (red dotted), 2016 (green dashed), 2017 (blue dash-dot) and 2018 (black solid).

Sea-surface temperature data from the global Operational Sea-Surface Temperature and Sea Ice Analysis (OSTIA) system (Donlon et al., 2012) was used to study the sea-surface temperature around England and Wales, indicated in Figs. 1 and 2. Using data from 2015 to 2017, a map of the average number of days per year when the sea-surface temperature is warmer than 18 °C was produced (Fig. 1a). Several regions in England and Wales where commercial shellfisheries exist were analysed further (Figs. 1 and 2). The time series sea-surface temperature for these locations are in Fig. 2 and a map of location of these sites is shown in Supplementary Data 1. We identified Chichester Harbour, Whitstable Bay and Osea Island as areas were sea-surface temperatures were favourable for *Vibrio* Species presence. Lyme Bay was identified as areas where sea-surface temperature were lower and unfavourable for growth of *Vibrio* species. Fig. 2 shows the time series of daily sea-surface temperature at these four locations between 2015 and 2018 as well as several other sites in Britain and Wales where commercial shellfisheries are based.

The sea-surface temperature at Whitstable Bay, Osea Island and Chichester Harbour during summer 2015 (June–August) was around 18 °C but peaked around 22 °C in these areas in 2018 (Fig. 2). In Lyme Bay, the sea-surface temperature remained stable, peaking at around ∼18 °C during the summer months, during this four year period (Fig. 2). The time series data plotted for Whitstable Bay, Osea Island, Chichester Harbour, Selsey, Thames Estuary and Cliff Pools show that in 2015 the temperature occasionally exceeded 18 °C (Fig. 2). Between 2016 and 2018 the sea-surface temperature at these sites had exceeded 18 °C and remained above this temperature for eight weeks or more consecutively. Although the observed the sea-surface temperature has increased in the areas mentioned above over the four-year period, the sea-surface temperature is variable around the coast of England and Wales and there is natural variability from year to year.

### 2.2 *Vibrio* abundance peaks in July and August

**Fig. 3:**
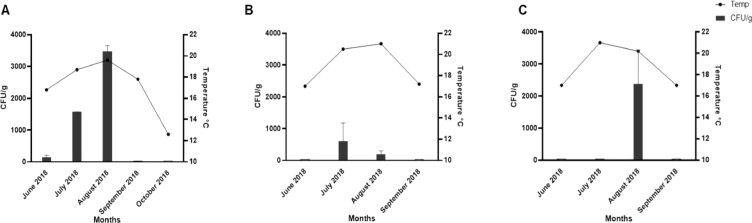
Total number of *Vibrio* species detected using direct plate enumeration from shellfish samples taken from a) Chichester Harbour b) Whitstable Bay c) Osea Island versus sea surface temperature. Samples were tested in duplicate (*n* = 2) and the average of both experiments is represented in datasets. Temperature data for Chichester Harbour is taken at the time of sampling at the site. Temperature taken for Whitstable Bay and Osea Island are taken from time series OSTIA sea-surface temperature values using a satellite/buoy composite.

Shellfish samples from Osea Island, Whitstable Bay, Chichester Harbour and Lyme Bay were examined from June-October in 2018 and total *Vibrio* species were recorded from plate counts of sucrose-positive and negative colonies on thiosulphate citrate bile sucrose agar (TCBS) plates before a further enrichment step. For depurated Pacific oysters from Whitstable Bay, the total numbers of *Vibrio* species peaked in July to 603 CFU/g (Fig. 3B, Table 1). For depurated Pacific oysters from the Osea Island area, the total numbers of *Vibrio* species peaked in August at 2384 CFU/g (Fig. 3C). Non-depurated blue mussels (*Mytilus edulis*) from Lyme Bay, where the sea-surface temperature only briefly rose above 18 °C (Fig. 2, Table 1), were also tested and found to be negative for *Vibrio* species between June and September 2018 unless an enrichment step was first performed. Human pathogenic *Vibrio* species such as *V. parahaemolyticus* and *V. vulnificus* were not identified from samples from Osea Island, Whitstable Bay or Lyme Bay.

**Table 1:** Shellfish samples tested in this study.

Sample number	Date of isolation	Site^a^	Type^b^	Total *Vibrio* species detected CFU/g	*Vibrio* species isolated
01/2018	June	B	A	< 10	*V. alginolyicus*
02/2018	June	C	A	< 10	*V. alginolyicus*
03/2018	June	D	B	0	*ND*
04/2018	June	A	C	140	*V. parahaemolyticus, V. alginolyicus*
05/2018	July	A	C	1573	*Photobacterium, V. jasicida, V. rotiferianus, V. alginolyicus*
06/2018	July	B	A	603	*V. jasicida, V. alginolyicus*
07/2018	July	C	A	15	*V. jasicida, V. alginolyicus*
08/2018	July	D	B	0	*ND*
09/2018	August	A	C	3480	*V. rotiferianus, V. jasicida, V. alginolyicus*
10/2018	August	B	A	198	*Photobacterium, V. rotiferianus, V. alginolyicus*
11/2018	August	C	A	2385	*V. jasicida, V. alginolyicus*
12/2018	August	D	B	0	*ND*
13/2018	September	A	C	25	*V. rotiferianus, V. jasicida, V. alginolyicus*
14/2018	September	B	A	< 10	*V. alginolyicus*
15/2018	September	C	A	< 10	*V. jasicida, V. alginolyicus*
16/2018	October	A	C	< 10	*V. jasicida, V. alginolyicus*

a _:_ Location of samples A – Chichester Harbour, B – Whitstable Bay, C – Osea Island, D – Lyme Bay,.

b : Type of sample A – *Crassostrea gigas*, B – *Mytilus edulis*, C – *Ostrea edulis* ND – Not determined.

For non-depurated European flat oysters (*Ostrea edulis*) samples from Chichester Harbour the total number of *Vibrio* species isolated in June when the temperature taken at site was an average of ∼16–17 °C, was < 140 CFU/g (Fig. 3 and Table 1). This increased to 3480 CFU/g in shellfish taken in August 2018, when the average temperature at the site was ∼20 °C (Fig. 3). A seasonal relationship was seen between increasing temperature and the total number of *Vibrio* species present in samples tested from Chichester Harbour for the five months of testing (r value of 0.90 using Spearman's Rho correlation) (Fig. 3a). *V. parahaemolyticus* was present at low levels in shellfish sample taken in June (sample EXE 04/2018 < 20 CFU/g) and representative isolates were confirmed as *V. parahaemolyticus* by PCR using the species marker toxR (Kim et al., 1999). The representative isolates tested were all negative for the TDH and TRH haemolysins known to be associated with virulence as determined by PCR for tdh and trh genes (Tada et al., 1992).

### 2.3 Biochemical characteristics of sucrose negative strains

One aim of this project was to measure the levels of the human pathogenic *Vibrio* species (*V. parahaemolyticus* or *V. vulnificus*) in shellfish from Whitstable Bay, Osea Island, and Chichester Harbour. Sucrose negative colonies on TCBS were further identified as presumptive V. parahaemolyticus or V vulnificus. Sucrose negative colonies were found in 10 of the 16 shellfish samples from Whitstable Bay (*n* = 2), Osea Island (*n* = 3), Lyme Bay (*n* = 0) and Chichester Harbour (*n* = 5). However, only one sample (EXE 04/2018) from Chichester Harbour, was positively identified further as *V. parahaemolyticus* (strain VP 04/2018). All other sucrose negative isolates were negative by PCR for *V. parahaemolyticus* species marker toxR (Kim et al., 1999) and tlh (Taniguchi et al., 1985) and *V. vulnificus* species marker vvhA (Hill et al., 1991). These sucrose negative isolates were Gram negative, oxidase positive, positive for fermentation of glucose, were not able to grow in 0% sodium chloride solution and grew well in solution containing > 2% sodium chloride. The results show that these isolates belong to the Vibrionaceae family.

The sucrose negative colonies on TCBS agar fell broadly into three groups depending on the biochemical tests applied. One group of isolates, from the Chichester Harbour and Whitstable Bay samples (EXE 05/2018, EXE 09/2018, EXE 10/2018 and EXE 13/2018) were positive for the production of indole from tryptophan and positive for the fermentation amygdalin (glycoside) (Table 2). These isolates were designated as Group A. Another group of sucrose negative colonies were designated Group B and were negative for indole production and negative for the fermentation of amygdalin. Group B isolates came from samples collected from Chichester Harbour and Osea Island. Finally, Group C included two isolates (EXE 05/2018 and EXE 10/2018) that were positively identified as *Photobacterium* species with a 99.8% API identification.

**Table 2:** Sequenced strains used in this study.

Strain number	Species	PCR tests						Biochemical tests			
		Group	ToxR	Tlh	TDH	TRH	Vvh	Oxidase	Glucose	Indole	Amygdalin
04/2018	*V. parahaemolyticus*	–	+	+	–	–	–	+	+	+	+
05/2018–4	*Photobacterium Species*	–	–	–	–	–	–	+	+	–	–
05/2018–10	*V. jasicida*	B	–	–	–	–	–	+	+	–	–
05/2018–5	*V. rotiferianus*	A	–	–	–	–	–	+	+	+	+
07/2018–1	*V. jasicida*	B	–	–	–	–	–	+	+	–	–
07/2018–7	*V. jasicida*	B	–	–	–	–	–	+	+	–	–
09/2018–4	*V. jasicida*	B	–	–	–	–	–	+	+	–	–
09/2018–7	*V. rotiferianus*	A	–	–	–	–	–	+	+	+	+
09/2018 - 11	*V. rotiferianus*	A	–	–	–	–	–	+	+	+	+
10/2018–1	*V. rotiferianus*	A	–	–	–	–	–	+	+	+	+
10/2018–4	*V. rotiferianus*	A	–	–	–	–	–	+	+	+	+
16/2018–1	*V. jasicida*	B	–	–	–	–	–	+	+	–	–
16/2018–2	*V. jasicida*	B	–	–	–	–	–	+	+	–	–
16/2018–3	*V. jasicida*	B	–	–	–	–	–	+	+	–	–

### 2.4 Whole genome sequencing analysis identifies *V. rotiferianus* and *V. jasicida* present in shellfish samples

**Fig. 4:**
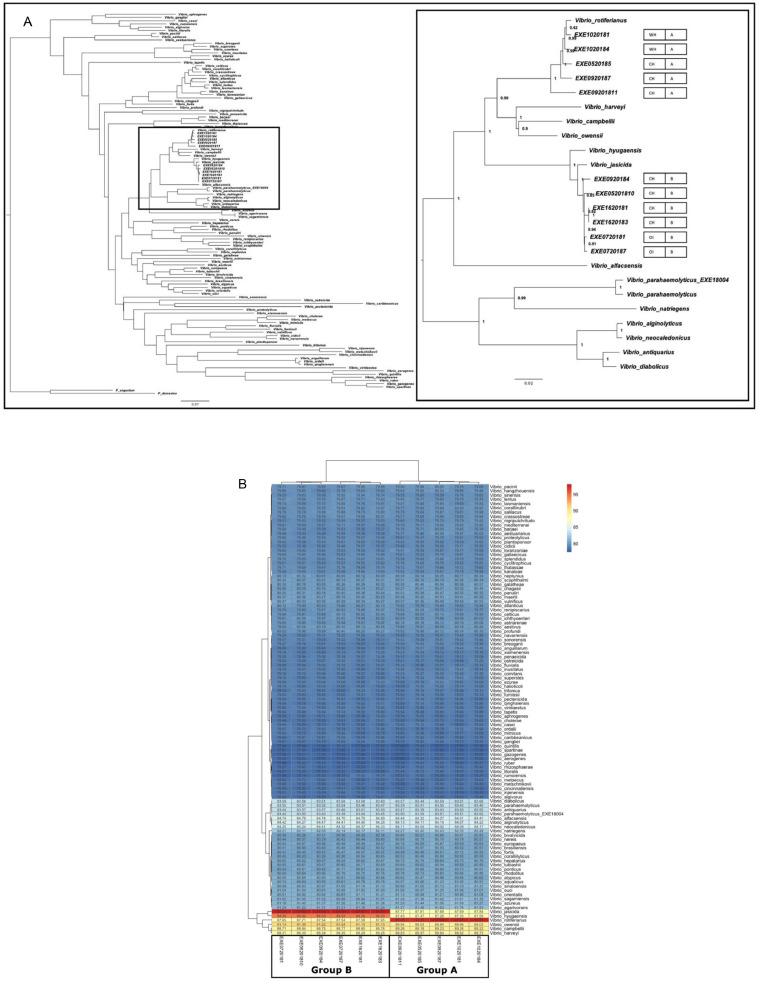
MLSA phylogeny of Group A and Group B strains. A - Multi Locus sequence analysis to determine the phylogenetic positions of the sequenced *Vibrio* isolates from Group A and Group B. Neighbour joining tree was constructed based on alignments of homologous sequences of 119 conserved ORFs. Scale bar represents 0.07 substitutions per site. B – Average nucleotide (ANI) analysis between genomes, with highest ANI observed for Group A strains as *V. rotiferianus* (>97%) and Group B as *V. jasicida* (> 97%).

Whole genome sequencing was carried out on 14 of the isolates collected. EXE 04/2018 (thought to be *V. parahaemolyticus*), five representative *Vibrio* isolates from Group A, six representative *Vibrio* isolates from Group B (Table 2) and one representative *Photobacterium* isolate from Group C). Initial MLSA results confirmed EXE 04/2018 as *V. parahaemolyticus* (Witherall et al., 2019) and indicated that Group A and Group B isolates fell into the *V. harveyi* clade grouping with *V. rotiferianus* (Group A) and *V. jasicida* (Group B). To confirm the identify of Group A and Group B to species level, average nucleotide identity (ANI) was calculated between these strains and publicly available *Vibrio* strains (Supplementary Data 2) (Fig. 4a). The highest ANI was observed between the isolates of Group A and *V. rotiferianus* B64D1 (97%) and secondly with Group B and *V. jasicida* 090810c (97%), both of which are above the hypothesised species delineation threshold value of 95% (Richter and Rossello-Mora, 2009). The MLSA and ANI analyses confirm that isolates sequenced in Group A were *V. rotiferianus* while isolates sequenced in Group B were *V. jasicida*.

**Fig. 5:**
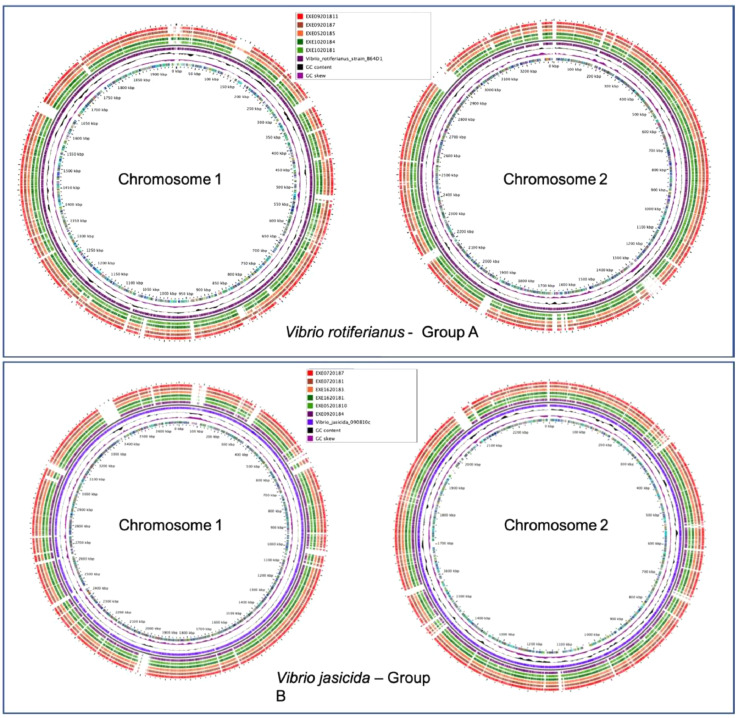
Genome comparison of *V. rotiferianus* and *V. jasicida*. A – Genome comparison of 5 *V. rotiferianus* strains (Group A) compared to available reference *V. rotiferianus* B64D1 strain. B – Genome comparison of 6 *V. jasicida* strain (Group B) compared to available reference *V. jasicida* 090810c strain. The alignments are visualized using Gview (Petkau et al., 2010). The innermost ring indicates the position on the reference chromosome. Positions covered by BLASTN alignments are indicated with a solid colour; whitespace gaps represent genomic regions not covered by the BLASTN alignments.

Fig. 5a shows circular genome maps comparing the assemblies of each sequenced *V. rotiferianus* stains in Group A aligned against that of *V. rotiferianus* B64D1 (GenBank accession number: CP018311.1) while Fig. 5b shows a circular genome map comparing assemblies of each sequenced *V. jasicida* stain in Group B aligned against that of *V. jasicida* 090810c (Genbank accession number GCA_002887615.1). These alignments both agree with the MLSA and ANI results. They show there is a high level of similarity between the strains from this study and the relevant reference strains used. However, these data do show that there is genomic variation within the *V. rotiferianus* clade and similarly within the *V. jasicida* clade.

**Fig. 6:**
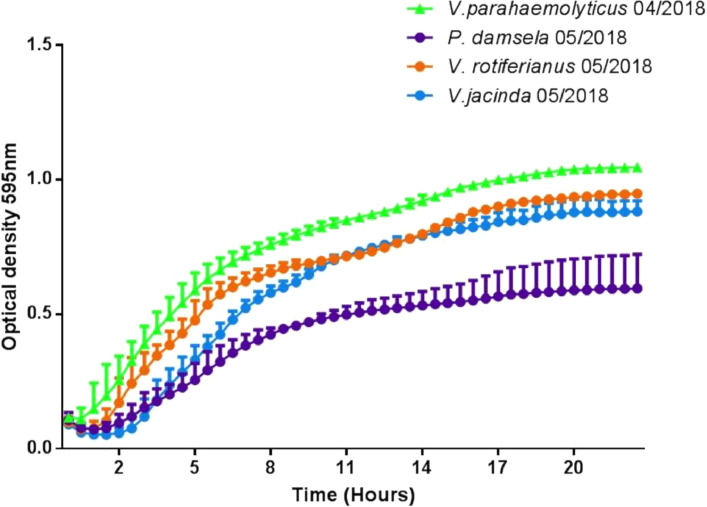
Growth curve of representative strains isolated from native oysters (*Ostrea edulis*) harvested from Chichester Harbour. Strains were tested with six technical repeats and two experimental repeats. Data shown is the average of both experiments with mean error.

Overall, *V. rotiferianus* was found in four of the 16 samples tested and was isolated from two locations tested in the UK in 2018 (Chichester Harbour and Osea Island). *Vibrio jasicida* was found in eight out of the 16 samples tested in this study and were also isolated from two locations in the UK in 2018 (Chichester Harbour and Whitstable Bay) (Table 1). Biochemically the only way to distinguish between *V. rotiferianus* and *V. jasicida* isolates was on the production of indole from tryptophan and the fermentation amygdalin (glycoside) (*V. rotiferianus* positive and *V. jasicida* negative). The growth of *V. rotiferianus* and *V. jasicida* was tested at 30 °C in Marine Broth and compared to growth of *V. parahaemolyticus* strains to determine and characteristic growth phenotypes. The *V. parahaemolyticus* strain 05/2018 grew at similar rates to reference RIMD2210633 (Fig. 6). Compared to the *Vibrio* strains tested, the growth of *Photobacterium damsela* in Marine Broth was slower (Fig. 6).

### 2.5 Pathogenic potential of *V. parahaemolyticus*

**Fig. 7:**
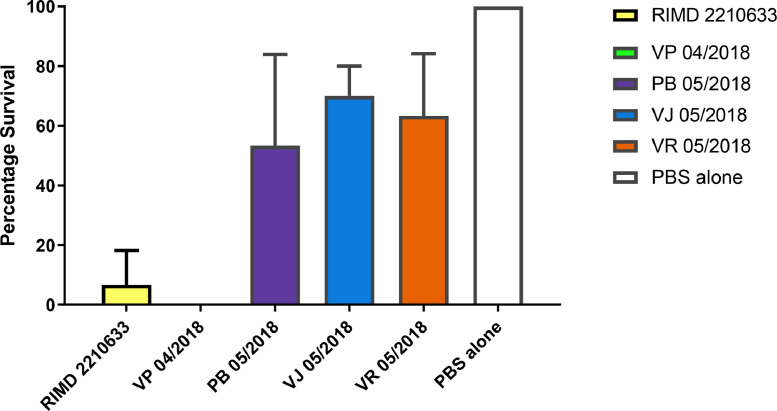
*Galleria mellonella* infection with strains isolated from native oysters (*Ostrea edulis*) harvested from Chichester Harbour. Stains were grown at 37 °C and a dose of 10^6^ CFU of each strain was injected into the larvae. Percentage survival was measured after 48 h. The results shown here are the means of three experiments, each using groups of 10 larvae paper strain. The error bars indicate standard deviation.

Previous work has shown that the insect infection model *Galleria mellonella* can be used to assess the virulence of *V. parahaemolyticus* strains (Wagley et al., 2017) and could distinguish clinical toxigenic strains from non-toxigenic strains from the environment. The *V. parahaemolyticus* strain RIMD 22,106,333, (a typical and well-referenced clinical TDH positive reference strain) was compared with the *V. parahaemolyticus* strain VP 05/2018 isolated from sample EXE 04/2018. Strain VP 04/2018 was negative for both TDH and TRH haemolysin genes. Both RIMD 2,210,633 and VP 04/2018 caused lethal infections at a dose of 10^6^ CFU per larvae (Fig. 7). The LD_50_ for RIMD2210633 was 10,000 CFU per larvae while the LD_50_ dose for EXE 04/2018 was 1000 CFU per larvae when the experiment was carried out at 37 °C indicating that VP 04/2018 was more virulent than the reference strain even without the presence of known virulence genes (*tdh* and *trh*) needed for human infection.

### 2.6 Pathogenic potential of *V. rotiferianus, V. jasicida* and *Ph. damsela*

Environmental strains *Ph. damsela* (PB 05/2018), *V. jasicida* (VJ 05/2018) and *V. rotiferianus* (VR 05/2018) were injected into *G. mellonella* at a dose of 10^6^ CFU per larvae. The results are shown in Fig. 7. Strain *Ph. damsela* (PB 05/2018) showed 50% survival while *V. jasicida* (VJ 05/2018) and *V. rotiferianus* (VR 05/2018) showed 70% and 60% survival, respectively at 10^6^ CFU per larvae. At a higher dose of 10^7^ CFU per larvae, VJ 05/2018 and VR 05/2018 showed 40% survival after 48 h (data not shown).

## 3 Discussion

One of the potential consequences of climate change will be changed patterns of parasite and microbial disease in aquaculture settings. One concern is the potential for an increased incidence of Vibriosis - diseases caused by *Vibrio* species. Vibriosis caused by some species, such as *V. parahaemolyticus*, can occur in humans causing gastroenteritis originating from raw or undercooked seafood. Vibriosis is also one of the main bacterial diseases of larvae and juvenile bivalves, affecting the early development stages of shellfish growth. The presence of *Vibrio* species, and their growth, is dependant on the water temperature of the environment. Thus, an increase in sea surface temperatures could lead to increased bacterial growth, leading to more disease.

In this study, sea-surface temperature data were assessed in several nearshore ecosystems in English and Welsh waters that accommodate UK shellfisheries (Fig. 2 and Supplementary Data 1). In several areas, an increase in peak summer sea-surface temperature from 18 °C to 22° was recorded over this four-year period. Furthermore, for some of these shellfish harvesting areas, the sea-surface temperature remained above 18 °C for up to eight consecutive weeks. Fluctuations at shellfish sites cannot be ruled out when comparing to sea-surface temperatures of wider regions however, temperature increases for prolonged periods could alter habitats and change the distribution of bacterial species within them. *Vibrio* species are predominantly found in locations where the temperatures are above 18 °C, a temperature that is suitable for their growth and proliferation. Increased sea-surface temperatures in nearshore ecosystems in the UK could allow for favourable growth conditions that support the growth of *Vibrio* species. Research carried out in the UK has mainly focussed on the presence of human pathogenic *Vibrio* species, such as *V. parahaemolyticus*, in the UK marine environment. These have been readily detected in some nearshore ecosystems since 2001 (Baker-Austin et al., 2010; Ford et al., 2020b; Martinez-Urtaza et al., 2013; Powell et al., 2013; Wagley et al., 2009, 2008), thus it is unsurprising that *V. parahaemolyticus* was detected in samples from Chichester Harbour when the temperature was suitable for bacterial growth. However, why *V. parahaemolyticus* was not detected in later months, when sea-surface temperature continued to be suitable for growth suggests that other factors such as competition from other *Vibrio* species and environmental abiotic factors in the community play a vital role in *V. parahaemolyticus* establishment. Efforts to use temperature and salinity data alone to predict the occurrence of pathogenic *Vibrio* species in an ecosystem have not always been reliable indicators of *Vibrio* emergence (McLaughlin et al., 2005). In some disease outbreaks, *Vibrio* species have appeared in shellfish where waters were less than < 18 °C, indicating that simplistic environmental conditions alone are not reliable indicators of disease prediction (McLaughlin et al., 2005). Conversely, in this study, increasing temperatures in nearshore ecosystems did not necessarily drive the occurrence of *V. parahaemolyticus* in the shellfish. Thus, further research is required to combine key environmental, biological, and molecular factors to ascertain the drivers behind the seasonal occurrence of *V. parahaemolyticus* in the natural environment.

During this study, our focus was on detecting the main human pathogens such as *V. parahaemolyticus* and *V. vulnificus*. However, during shellfish testing, *V. alginolyticus* was also detected in nearly all shellfish samples and can be considered part of normal marine flora (Xie et al., 2005). It should be noted that *V. alginolyticus* can increase health risk for consumers (Baker-Austin et al., 2018; Reilly et al., 2011) and further studies need to be conducted to ascertain whether *V. alginolyticus* strains from the UK are a risk to consumers and recreational users of waterways where *V. alginolyitcus* are widely present.

This study shows that members of the *V. harveyi* clade are also present in nearshore ecosystems in the UK and that *Vibrio* species may play a role in both human and shellfish associated diseases. The core of the *V. harveyi* clade is made up of *V. harveyi* itself and closely related species including *V. alginolyticus, V. azureus, V. mytili, V. natriegens, V. campbellii, V. parahaemolyticus, V. owensii, V. jascinda, V. sagamiensis* and *V. rotiferianus*. The members of *V. harveyi clade* share a high degree of genetic and phenotypic similarity, which can often lead to the misidentification of species within this clade (Gomez-Gil et al., 2004; Lin et al., 2010; Sawabe et al., 2007). The analysis of shellfish samples from the UK in the summer of 2018 identified the presence of *V. parahaemolyticus, V. jasicida*, and *V. rotiferianus* in three shellfish sites around the UK. To the best of our knowledge, this study is the first to identify and report *V. rotiferianus* and *V. jasicida* presence in shellfish harvesting areas in England. Previous testing of shellfish (prior to 2008) for the presence of *Vibrio* species in UK sites did not detect the presence of *V. rotiferianus* and *V. jasicida* in UK shellfisheries (Wagley et al., 2008). *V. rotiferianus* was first described by Gomez-Gil and co-workers in 2003, isolated from the rotifer *Brachionus plicatilis* (Gomez-Gil et al., 2003). This species was described as growing yellow sucrose positive colonies on TCBS agar and was positive for the production of indole. In this study, *V. rotiferianus* strains isolated were positive for indole production but grew as sucrose negative (green) colonies on TCBS agar indicating that these strains are biochemically different from those first described in the literature. *V. jasicida*, first described in 2012, has been recovered from marine invertebrates and vertebrates, including Atlantic salmon and flounder (Yoshizawa et al., 2012). In this study, we examined the pathogenic potential of the *V. jasicida* and *V. rotiferianus* in an insect infection model using *G. mellonella* larvae. This model has been shown to be a useful for studying human pathogenic *Vibrio* species as well as aquaculture *Vibrio* and non-*Vibrio* species (Djainal et al., 2020; McMillan et al., 2015). The data here indicate that the strains of *V. jasicida* and *V. rotiferianus* tested are able to cause infection and mortality in *G. mellonella*, and this model could be used in the future to further investigate the virulence of these aquaculture pathogens. Members of the *V. harveyii* clade are a major concern for sustainability of the aquaculture industry (King et al., 2019; Vezzulli et al., 2010; Wendling et al., 2014), thus, it is vital to understand the epidemiological significance of these new emerging *Vibrio* species before any associated disease burden becomes a problem in aquaculture settings.

**Using a ‘One Health’ aquaculture approach is needed to understand the decline in native oysters at Chichester Harbour.**

Chichester Harbour is home to the native oysters (*Ostrea edulis*) and once supported a substantial fishery, but stocks of native oysters have declined significantly over the last 20 years (Helmer et al., 2019). Globally, 85% of all oyster beds and reefs have been lost, and across Europe and England up to 95% of native oyster reefs have been extirpated (Airoldi and Beck, 2007; Beck et al., 2011). This trend has been reflected in the Solent, once home to one of Europe's largest native oyster fisheries, with seabed densities of 32m^2^, supporting 450 commercial vessels in the 1980′s (Key and Davidson, 1981). Native oyster stocks have since declined, suffering a population crash in 2007 from which they have not recovered, and remain severely depleted in many harbours, including Chichester Harbour. A long-term study found numbers of *O. edulis* within Chichester Harbour had decreased by 96%, between 1998 and 2017 (Helmer et al., 2019). Sussex Inshore Fisheries and Conservation Authority (IFCA) has been conducting annual stock assessments (catch per unit effort (CPUE) estimates) for the oysters in Chichester Harbour since 2013. In the main fishery area (Emsworth Channel), the CPUE decreased from 50 kg/h/1 min dredge width in 2013 to 22 kg/h/1 min in 2017. In 2018, the CPUE dropped to 3.9 kg/h/1 min and the fishery was closed. CPUE remained low in 2019 (1.4 kg/h/1 min) and the fishery has remained closed. The continued low CPUE levels indicates a lack of continued oyster recruitment in Chichester Harbour and efforts to restore the populations of native oysters have been unsuccessful in recent years.

Several reports of the implications of *Vibrio* species acting as a potential disease causing agent involved in oyster mortality have been published (King et al., 2019; Waechter et al., 2002; Wendling et al., 2014) but in the majority of records no clear aetiological agent can be identified (Garnier et al., 2007). In this study, the presence of *Vibrio* species such as *V. jasicida*, and *V. rotiferianus* belonging to the *V. harveyi* clade is reported and this may play a role in oyster disease in this area. Further contributions to disease in oysters may be attributed to abiotic factors such as temperature, nutrient concentrations, and salinity as well as host oyster genetic backgrounds that make them sensitive to disease (de Lorgeril et al. 2018; Lang et al., 2010; Malham et al., 2009; Samain et al., 2007). The low levels of oyster recruitment in Chichester Harbour may also be due to the level of harvesting exceeding the reproductive capacity of the species as well as the presence of the invasive slipper limpet (*Crepidula fornicate*) that can outcompete *O. edulis* larvae leading to a dominance of *C. fornicata* in the Solent (Preston et al., 2020). Leakage of partially treated sewage into Chichester Harbour between 2010 and 2015 may also have contributed to the low levels of oyster recruitment in the harbour, which needs to be further investigated (Environment Agency v Southern Water Services Ltd (2021) All ER (D) 62 (Aug)). Furthermore, oysters are typically populated with multiple bacterial and viral species. *In vivo* infection studies to find the causative agents of oyster mortality do not consider the complexity in the natural environment or how microorganisms interact with each other to cause host disease.

Bivalve larvae can naturally exhibit a high mortality during their transition to a juvenile state. Whether the *Vibrio* strains isolated in this study are responsible for bivalve larvae death in Chichester Harbour requires more investigation. Metagenomic time-series studies targeting the microbiome of oysters and the surrounding environments coupled with a survey of environmental abiotic factors and host susceptibility such as those conducted by King and co-workers to study the mortality events in the Pacific oyster in Australia (King et al., 2019) are needed in this area to identify causes of oyster mortality events. To understand why native oysters populations have declined in Chichester Harbour, it is thus important to use a ‘One Health’ approach (Stentiford et al., 2020). This approach examines all hazards that may contribute to water quality and the health of the oysters if aquaculture is going to thrive again in Chichester Harbour. These hazards may include chemicals such as naturally occurring biotoxins and partially treated sewage leaks entering the harbour, as well as the presence of increasing numbers and species of animal and human pathogens.

## 4 Conclusion

This study assessed the presence of culturable bacterial species in shellfish from multiple sites around England and found them to be populated with multiple *Vibrio* and non-*Vibrio* species. A number of factors could explain the increasing amount of *Vibrio* species now present in temperate regions including the UK marine and estuarine environments. Firstly, favourable growth conditions for *Vibrio* species such as increasing sea-surface temperatures and heavy rainfall events during the summer, (which can bring the water salinity down to optimum), are currently present in some areas around the UK (Baker-Austin et al., 2018, 2013). Secondly, globalisation and the importing of more seafood from regions that have a higher incidence of *Vibrio* species in the environment may favour the introduction of a variety of species into our waters. Connected to this is the increase in shipping and the release of ship ballast water that has been shown to be a transport vector for *Vibrio* species (Drake et al., 2007; Mimura et al., 2005; Rivera et al., 2012; Ruiz et al., 2000). If ballast water is contaminated with *Vibrio* species and is discharged into harbours and coastal waterways then this could lead to the introduction and establishment of new pathogenic and non-pathogenic *Vibrio* species into the environment. Finally, the improvement of detection methods of *Vibrio* species from water and shellfish in the UK has improved vastly over the past 15 years and in the advent of cheaper and faster genome sequencing, scientists are able to sequence strains that we previously may have overlooked. Consequentially, the conditions that favour the growth and establishment of *Vibrio* species as a seasonal human and aquaculture pathogen in temperate regions, is a public health concern and should be monitored in the environment.

## 5 Methods and materials

### 5.1 Shellfish collection and bacterial isolation

Bacterial strains used in this study are shown in Table 1. *V. parahaemolyticus* strains were initially cultured aerobically onto selective media Thiosulphate Citrate Bile Sucrose (TCBS) agar (Oxoid) at 37 °C for 24 h. For enumeration of colony counts, routine sub culturing and growth, Marine Agar (Conda Labs, Spain) was used and incubated at 30 °C for 24 h.

For shellfish testing, non-depurated samples were sought initially from all sites but could only be obtained from Chichester Harbour and Lyme Bay while commercially depurated shellfish were obtained from Osea Island and Whitstable Bay. In the five months of June to October 2018, a total of five shellfish samples were tested for the presence of *Vibrio* species from Chichester Harbour. Shellfish were supplied as part of ongoing research programmes and were received confidentially directly from harvesting areas. Samples comprised of non-depurated *O. edulis* and were held between 4 and 8 °C during transit and, on arrival in the laboratory, were placed in the fridge until analysis. Further, commercially available shellfish samples that had been harvested from Osea Island and Whitstable Bay and non-depurated *M. edulis* from Lyme Bay were also obtained and tested for the presence of *Vibrio* species. Samples were tested in duplicate and the average of both experiments was used to calculate the total *Vibrio* species count. All raw shellfish samples were analysed within 24 h of collection and according to ISO 21,872–1 with minor modifications. A total of 25 g of shellfish meat (taken from a minimum of eight animals) was disrupted using a stomacher before the addition of 225 ml of alkaline salt peptone water (ASPW; Oxoid Ltd., Basingstoke, Hampshire, UK). Sequential log dilutions of stomached shellfish meat were carried out in APSW to a maximum of 10^−1^ and 100 µl was spread onto the surface of TCBS plates to determine direct enumeration of *Vibrio* species. Total *Vibrio* species were recorded from plate counts of sucrose-positive and negative colonies on thiosulphate citrate bile sucrose agar (TCBS) plates before a further enrichment step. All samples were then incubated at 41 °C for 6 h for an enrichment step, after which a 5 µl loopful was taken from directly below the surface of the broth and streaked onto TCBS plates. All TCBS plates were incubated at 37 °C for 24 h. Typical sucrose negative (green) colonies were subcultured onto marine agar (Conda labs, Spain) and incubated at 30 °C for 24 h. Presumptive colonies were identified as *V. parahaemolyticus* if they met the following criteria: positive for oxidase, negative for Voges Proskauer and Ortho-nitrophenyl-β-d-galactopyranoside, no growth in 0% NaCl and no acid from sucrose. Further identification by API 20E strips (BioMerieux) was also carried out on sucrose negative colonies. All biochemically identified *V. parahaemolyticus* strains were further analysed by PCR amplification using the species target toxR (Kim et al., 1999). All sucrose negative colonies that were also negative for *V. parahaemolyticus* PCR tests were checked for *V. vulnificus* identification using PCR amplification using the species target vvh (Hill et al., 1991).

### 5.2 Genome sequencing, assembly and annotation

Thirteen unidentified strains and one *V. parahaemolyticus* strain (Table 1) were genome sequenced. Bacterial cells were harvested and genomic DNA was extracted using Wizard Genomic DNA Purification Kit (Promega). Library preparation was carried out by Exeter DNA sequencing service. In brief, DNA was concentrated using GeneRead kit (Lot No. 145,025,210) and end repair and adenylation of fragments was carried using NEXTFLEX® Rapid DNAseq kit (#5144–02) according to manufactures instructions. Purification and concentration of PCR amplified library was carried out according to GeneRead kit instructions. Genome wide sequence data (150 bp, paired end) was generated using an Illumina NovaSeq. Raw sequencing reads were screened for contamination and quality filtered using Trimmomatic (Bolger et al., 2014). Sequencing metrics were generated using FastQC (Wingett and Andrews, 2018). This filtered dataset was then assembled de-novo using SPADES v3.15.1 (Bankevich et al., 2012). De-novo assemblies were assessed for completeness using BUSCO (Seppey et al., 2019) (Supplementary Data 3) and annotated using PROKKA (Seemann, 2014). Assembly metrics were generated using the QUAST (Gurevich et al., 2013).

### 5.3 Multi Locus sequence analysis (MLSA)

Multi Locus sequence analysis (MLSA) was conducted on all sequenced strains and a panel of reference strains downloaded from the NCBI genomes database (Feb 2021). The MLSA was conducted using six conserved housekeeping genes (efp, glnA, gyrB, metG, purM, pntA). The sequences of these genes were extracted from the genome annotations for each strain and concatenated. The concatenated MLSA sequences were aligned using MUSCLE (Edgar, 2004) and phylogenetic analysis was conducted using MEGA 10 (Kumar et al., 2016). Maximum likelihood trees were generated using the general time reversible model and 100 bootstrap replicates were performed. *Photobacterium* species were initially used as the outgroup to root the tree.

### 5.4 Evolutionary analysis by maximum likelihood method

The evolutionary history was inferred by using the Maximum Likelihood method and General Time Reversible model (Nei and Kumar, 2000). The tree with the highest log likelihood (−291,299.34) is shown in Fig. 4a. Initial tree(s) for the heuristic search were obtained automatically by applying neighbour-Join and BioNJ algorithms to a matrix of pairwise distances estimated using the Maximum Composite Likelihood (MCL) approach, and then selecting the topology with superior log likelihood value. A discrete Gamma distribution was used to model evolutionary rate differences amongst sites (five categories (+G, parameter = 0.7879)). The rate variation model allowed for some sites to be evolutionarily invariable ([+I], 45.80% sites). The tree is drawn to scale, with branch lengths measured in the number of substitutions per site. This analysis involved 119 nucleotide sequences. Codon positions included were 1st+2nd+3rd+Noncoding. All positions with less than 95% site coverage were eliminated, i.e., fewer than 5% alignment gaps, missing data, and ambiguous bases were allowed at any position (partial deletion option). There was a total of 8965 positions in the final dataset. Evolutionary analyses were conducted in MEGA X (Kumar et al., 2018).

### 5.5 Average nucleotide identity (ANI)

Average nucleotide identity was calculated using fastANI (Jain et al., 2018) between the sequenced isolates and a reference panel of *Vibrio* genomes downloaded from the NCBI genomes database (February 2021).

### 5.6 Determining sea surface temperatures for UK coastal waters

The sea-surface temperature data is a daily value, valid at foundation depth. This is the depth, several meters down, where there are no variations on the sub-daily timescale and hence can be treated as a daily-mean value. The data is from the Operational Sea Surface Temperatures and Sea Ice Analysis (OSTIA), which is used to provide the sea-surface temperature in Met-Office global weather forecasts. The sea temperature dataset is produced by combining observations from satellite and buoys and filling gaps between these observations in a physically consistent manner. The time-series plotted in Fig. 2 were extracted for the locations nearest to the sample collections sites (Supplementary data 1). However, since the sea-surface temperature data are available on a 0.05 × 0.05 latitude-longitude grid, corresponding to roughly a 5 km x 5 km spatial average, the nearest location may be a couple of kilometres away from the sampling site. Additionally, they are more likely to be representative of the offshore sea-surface temperature than the temperature within a sheltered harbour. Despite these caveats, the OSTIA data can be used as a guide of where sea-surface temperatures are likely to be suitable for the presence of *Vibrio* species. During 2018, the sea-surface temperature plots were updated every few weeks to track the warming of the seawaters as summer approached and to help inform when to acquire the shellfish samples.

### 5.7 Infection of Galleria mellonella larvae

*Galleria mellonella* larvae were purchased from TruLarv™ (Biosystems Technology, Exeter, Devon, UK). Larvae weighing between 0.2 and 0.35 g were chosen for experiments. For each experiment a total of ten larvae were used per strain to be tested. The larvae were infected by micro-injection (Hamilton Ltd) into the right foremost proleg with 10^6^ CFU per larvae of *V. parahaemolyticus* in 10 µl volume which had been grown in Marine Broth at 37 °C and washed twice in PBS. Bacterial cell counts were carried out by plating serial dilutions of the inoculum onto Marine agar. For control purposes, ten larvae were inoculated with PBS and a further ten were left inoculated. The larvae were incubated at 37 °C and survival was recorded for all strains after 24 and 48 h. Larvae were scored as dead when they ceased moving or failed to respond when gently manipulated with a pipette tip. Observation findings were also recorded if larvae changed colour from their normal pale cream coloration to brown or black indicative of melanisation.

## Declaration of Competing Interest

The authors declare that they have no known competing financial interests or personal relationships that could have appeared to influence the work reported in this paper.

## Appendix Supplementary materials

Image, application 1

Image, application 2

Image, application 3
