# Supplementary figures and images for: The increased prevalence of Vibrio species and the first reporting of Vibrio jasicida and Vibrio rotiferianus at UK shellfish sites
Source: Water Res. 2022 Mar 1;211:117942. doi: 10.1016/j.watres.2021.117942 (PMC8841665; doi:10.1016/j.watres.2021.117942)

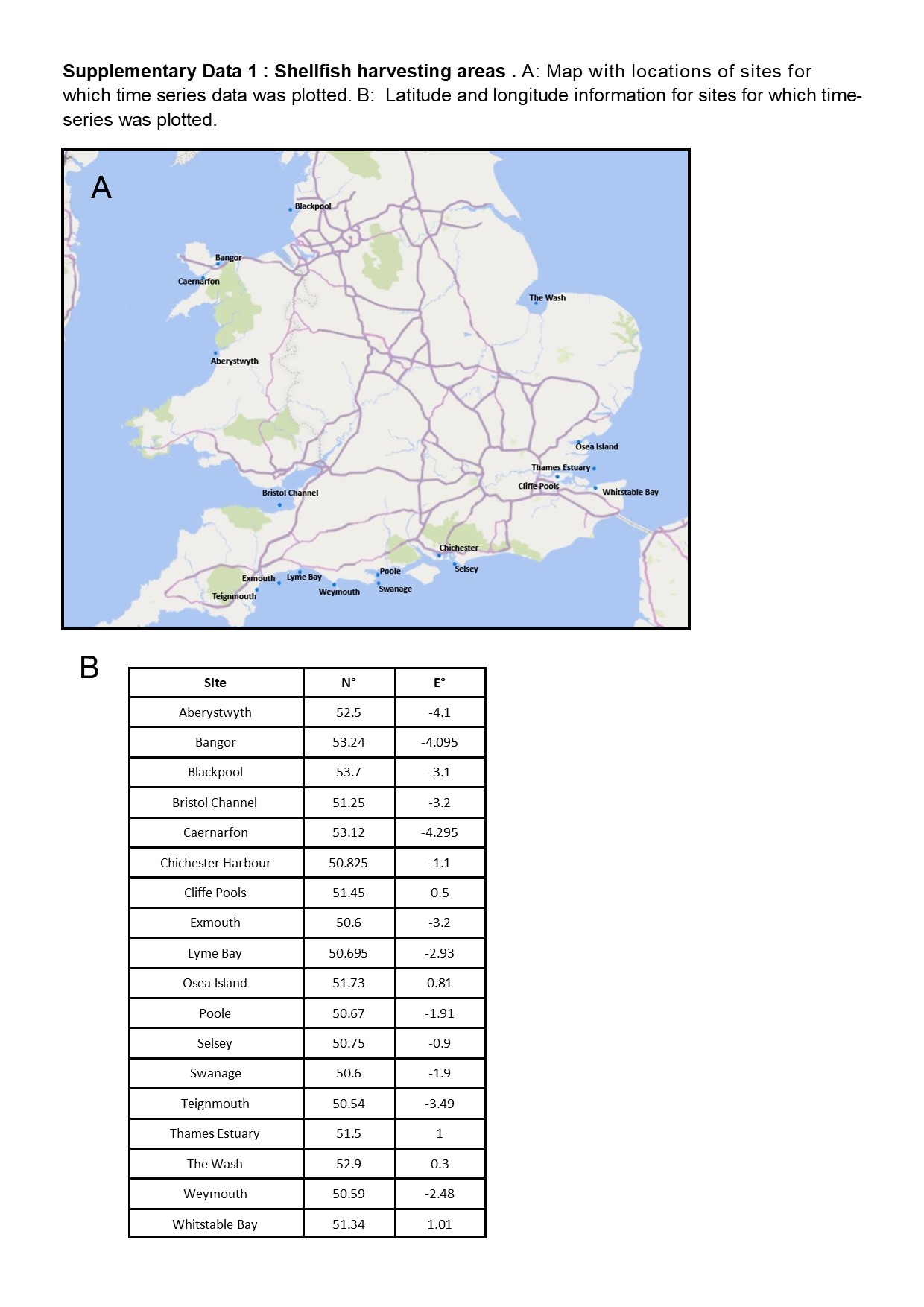

Supplement: Supplementary file 1 [file mmc1.zip › mmc1.jpg]

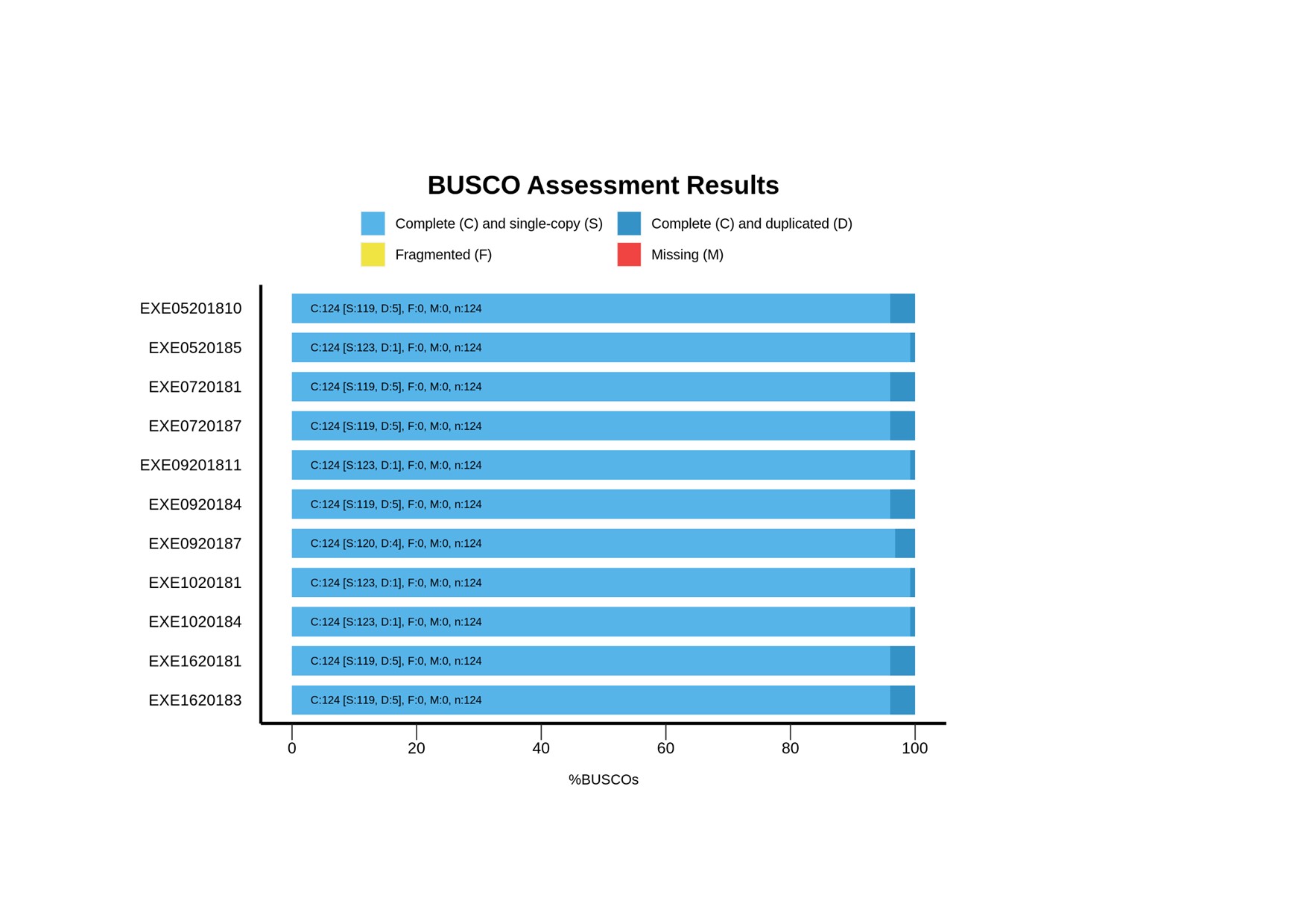

Supplement: Supplementary file 3 [file mmc3.zip › mmc3.jpg]
