# Supplementary material for: The increased prevalence of Vibrio species and the first reporting of Vibrio jasicida and Vibrio rotiferianus at UK shellfish sites
Source: Water Res. 2022 Mar 1;211:117942. doi: 10.1016/j.watres.2021.117942 (PMC8841665; doi:10.1016/j.watres.2021.117942)
Supplement: Supplementary file 2 [file mmc2.docx]

| Accession number | Genus | Species | Strain |
| --- | --- | --- | --- |
| NZ_FQXZ01000077.1 | Vibrio | aerogenes | CECT7868 |
| NZ_JYJN01000001.1 | Vibrio | aestivus | CAIM1861 |
| NZ_VTXH01000001.1 | Vibrio | aestuarianus | O-00-16-10 |
| NZ_POWH01000001.1 | Vibrio | agarivorans | CAIM1911 |
| NZ_CP032093.1 | Vibrio | alfacsensis | CAIM1831 |
| NC_022349.1 | Vibrio | alginolyticus | ATCC17749 |
| NZ_AP018678.1 | Vibrio | algivorus | NBRC111146 |
| NC_015633.1 | Vibrio | anguillarum | 775 |
| NC_013456.1 | Vibrio | antiquarius |  |
| NZ_AP018689.1 | Vibrio | aphrogenes | CA-1004 |
| NZ_RXZH01000001.1 | Vibrio | aquaticus | BEI207 |
| NZ_CP047475.1 | Vibrio | astriarenae | HN897 |
| NZ_FLQP01000100.1 | Vibrio | atlanticus | CECT7223 |
| NZ_BLID01000001.1 | Vibrio | atypicus | DSM25292 |
| NZ_BATL01000166.1 | Vibrio | azureus | NBRC104587 |
| NZ_LQXO02000001.1 | Vibrio | barjaei | 3062 |
| NZ_LLEI02000001.1 | Vibrio | bivalvicida | 605 |
| NZ_AEVS01000116.1 | Vibrio | brasiliensis | LMG20546 |
| NZ_CP016177.1 | Vibrio | breoganii | FF50 |
| NC_009783.1 | Vibrio | campbellii | ATCCBAA-1116 |
| NZ_AEIU01000126.1 | Vibrio | caribbeanicus | ATCCBAA-2122 |
| NZ_AP018680.1 | Vibrio | casei | DSM22364 |
| NZ_FLQZ01000185.1 | Vibrio | celticus | CECT7224 |
| NZ_CP034970.1 | Vibrio | chagasii | ECSMB14107 |
| NZ_CP010812.1 | Vibrio | cholerae | 10432-62 |
| NZ_CP046804.1 | Vibrio | cidicii | 2756-81 |
| NZ_CP046802.1 | Vibrio | cincinnatiensis | 2070-81 |
| NZ_BJLH01000001.1 | Vibrio | comitans | NBRC102076 |
| NZ_CP009617.1 | Vibrio | coralliilyticus | RE98 |
| NZ_LNRH01000001.1 | Vibrio | coralliirubri | MARo |
| NZ_CCJW01000001.1 | Vibrio | crassostreae | LGP7 |
| NZ_CP039700.1 | Vibrio | cyclitrophicus | ECSMB14105 |
| NZ_CP014036.1 | Vibrio | diabolicus | FDAARGOS_105 |
| NZ_LUAX01000001.1 | Vibrio | europaeus | PP-638 |
| NZ_BATM01000067.1 | Vibrio | ezurae | NBRC102218 |
| NZ_CP014034.2 | Vibrio | fluvialis | FDAARGOS_104 |
| NZ_JFFR01000001.1 | Vibrio | fortis | Dalian14 |
| NZ_CP040990.1 | Vibrio | furnissii | FDAARGOS_777 |
| NZ_JXXV01000001.1 | Vibrio | galatheae | S2757 |
| NZ_SZXU01000001.1 | Vibrio | gallaecicus | DSM23502 |
| NZ_PPSN01000001.1 | Vibrio | gangliei | SZDIS-1 |
| NZ_CP018835.1 | Vibrio | gazogenes | ATCC43942 |
| NZ_BAUJ01000109.1 | Vibrio | halioticoli | NBRC102217 |
| NZ_FNVG01000060.1 | Vibrio | hangzhouensis | CGMCC1.7062 |
| NZ_CP014038.2 | Vibrio | harveyi | FDAARGOS_107 |
| NZ_LHPI01000001.1 | Vibrio | hepatarius | DSM19134 |
| NZ_CP025794.1 | Vibrio | hyugaensis | 090810a |
| NZ_AFWF01000326.1 | Vibrio | ichthyoenteri | ATCC700023 |
| NZ_BDJF01000001.1 | Vibrio | injenensis | M12-1144 |
| NZ_BJLF01000001.1 | Vibrio | inusitatus | NBRC102082 |
| NZ_CP025792.1 | Vibrio | jasicida | 090810c |
| NZ_VZPY01000001.1 | Vibrio | kanaloae | CCUG56968 |
| NZ_MAKA01000001.1 | Vibrio | lentus | 5F79 |
| NZ_KE384566.1 | Vibrio | litoralis | DSM17657 |
| NZ_QRHC01000001.1 | Vibrio | maerlii | G62 |
| NZ_BCUE01000001.1 | Vibrio | mediterranei | NBRC15635 |
| NZ_CP046820.1 | Vibrio | metoecus | 2011V-1015 |
| NZ_ACZO01000011.1 | Vibrio | metschnikovii | CIP69.14 |
| NZ_CP014042.2 | Vibrio | mimicus | FDAARGOS_112 |
| NZ_CP016347.1 | Vibrio | natriegens | CCUG16371 |
| NZ_JMCG01000001.1 | Vibrio | navarrensis | ATCC51183 |
| NZ_CP032213.1 | Vibrio | neocaledonicus | CGJ02-2 |
| NZ_JXXU01000001.1 | Vibrio | neptunius | S2394 |
| NZ_BCUD01000001.1 | Vibrio | nereis | NBRC15637 |
| NC_022528.1 | Vibrio | nigripul |  |
| NZ_AEZC01000001.1 | Vibrio | ordalii | ATCC33509 |
| NZ_ACZV01000005.1 | Vibrio | orientalis | ATCC33934 |
| NZ_MPHM01000001.1 | Vibrio | ostreicida | UCD-KL16 |
| NZ_SATR01000001.1 | Vibrio | ouci | BEI176 |
| NZ_CP019959.1 | Vibrio | owensii | XSBZ03 |
| NZ_JONH01000001.1 | Vibrio | pacinii | DSM19139 |
| NZ_MJMJ01000001.1 | Vibrio | panuliri | CAIM703 |
| NC_004603.1 | Vibrio | parahaemolyticus | RIMD2210633 |
| Ga0364980_11 | Vibrio | Parahaemolyticus | EXE18/004 |
| NZ_RSFA01000001.1 | Vibrio | pectenicida | CAIM594 |
| NZ_BFAQ01000001.1 | Vibrio | penaeicida | TUMSAT-NU1 |
| NZ_JABEQC010000001.1 | Vibrio | plantisponsor | LMG24470 |
| NZ_AP019657.1 | Vibrio | ponticus | DSM16217 |
| NZ_RZIS01000001.1 | Vibrio | profundi | TP187 |
| NZ_BATJ01000050.1 | Vibrio | proteolyticus | NBRC13287 |
| NZ_CP022741.1 | Vibrio | qinghaiensis | Q67 |
| NZ_FRFG01000125.1 | Vibrio | quintilis | CECT7734 |
| NZ_JTKH01000001.1 | Vibrio | renipiscarius | DCR37347 |
| NZ_KL543967.1 | Vibrio | rhizosphaerae | DSM18581 |
| NZ_QLYZ01000001.1 | Vibrio | rhodolitus | G98 |
| NZ_CP018311.1 | Vibrio | rotiferianus | B64D1 |
| NZ_FULE01000103.1 | Vibrio | ruber | DSM16370 |
| NZ_AP018685.1 | Vibrio | rumoiensis | FERMP-14531 |
| NZ_BAOJ01000504.1 | Vibrio | sagamiensis | NBRC104589 |
| NZ_NIVQ01000010.1 | Vibrio | salilacus | DSG-S6 |
| NZ_CP016307.1 | Vibrio | scophthalmi | VS-12 |
| NZ_AEVT01000135.1 | Vibrio | sinaloensis | DSM21326 |
| NZ_QVMU01000001.1 | Vibrio | sinensis | BEI233 |
| NZ_LJJE01000001.1 | Vibrio | sonorensis | CAIM1076 |
| NZ_FSSB01000061.1 | Vibrio | spartinae | CECT9026T |
| NZ_CP031055.1 | Vibrio | splendidus |  |
| NZ_BCUR01000001.1 | Vibrio | superstes | G3-29 |
| NZ_LT960611.1 | Vibrio | tapetis subsp. Tapetis | CECT4600 |
| NZ_AJZQ02000001.1 | Vibrio | tasmaniensis | ZS-17 |
| NZ_OANU01000191.1 | Vibrio | thalassae | CECT8203 |
| NZ_LMXU01000001.1 | Vibrio | toranzoniae | Vb10.8 |
| NZ_AP014635.1 | Vibrio | tritonius | AM2 |
| NZ_CP009354.1 | Vibrio | tubiashii | ATCC19109 |
| NZ_RJVQ01000001.1 | Vibrio | viridaestus | LJC006 |
| NZ_CP014636.1 | Vibrio | vulnificus | CECT4999 |
| NZ_FNDD01000081.1 | Vibrio | xiamenensis | CGMCC1.10228 |
